# Surgical and Radiology Trainees’ Proficiency in Reading Mammograms: the Importance of Education for Cancer Localisation

**DOI:** 10.1007/s13187-023-02393-7

**Published:** 2023-12-15

**Authors:** J. B. Wells, S. J. Lewis, M. Barron, P. D. Trieu

**Affiliations:** Discipline of Medical Imaging Sciences, Faculty of Medicine and Health, University of Sydney, D18 Susan Wakil Health Building, Western Avenue, Camperdown, NSW 2006 Australia

**Keywords:** Radiology, Breast, Surgery, Cancer, Education

## Abstract

Medical imaging with mammography plays a very important role in screening and diagnosis of breast cancer, Australia’s most common female cancer. The visualisation of cancers on mammograms often forms a diagnosis and guidance for radiologists and breast surgeons, and education platforms that provide real cases in a simulated testing environment have been shown to improve observer performance for radiologists. This study reports on the performance of surgical and radiology trainees in locating breast cancers. An enriched test set of 20 mammography cases (6 cancer and 14 cancer free) was created, and 18 surgical trainees and 32 radiology trainees reviewed the cases via the Breast Screen Reader Assessment Strategy (BREAST) platform and marked any lesions identifiable. Further analysis of performance with high- and low-density cases was undertaken, and standard metrics including sensitivity and specificity. Radiology trainees performed significantly better than surgical trainees in terms of specificity (0.72 vs. 0.35; *P* < 0.01). No significant differences were observed between the surgical and radiology trainees in sensitivity or lesion sensitivity. Mixed results were obtained with participants regarding breast density, with higher density cases generally having lower performance. The higher specificity of the radiology trainees compared to the surgical trainees likely represents less exposure to negative mammography cases. The use of high-fidelity simulated self-test environments like BREAST is able to benchmark, understand and build strategies for improving cancer education in a safe environment, including identifying challenging scenarios like breast density for enhanced training.

## Introduction

In Australian women, breast cancer has the highest cancer incidence, with approximately 20,000 new cases being diagnosed in 2022.^1^ According to the National Mortality Database (NMD), there have been over 3000 Australian deaths due to breast cancer since 2019 [1]. While the 5-year breast cancer survival rate in females is 92%, this is highly dependent on the stage at which the breast cancer is diagnosed, being close to 100% survival for Stage 1, however only 32% for Stage 4. As such, early detection of breast cancer via regular screening is imperative in reducing breast cancer mortality and increasing survival rates. This need is recognised by the creation of the national population-based screening programme BreastScreen Australia (BSA), which screens women aged 50–74 years old for breast cancer every 2 years using mammography.

Breast cancer treatment options vary depending on the type and stage of breast cancer and the patient’s condition. Interventions such as surgical resection form the basis of definitive treatment in 60–80% of Australian women with early-stage breast cancer and is normally followed by adjuvant therapy [2]. Surgical methods for breast cancer excision can be divided into breast-conserving procedures and mastectomy. Breast-conserving surgery includes lumpectomy, partial mastectomy, or wide local excision with safety margins. In Australia, approximately 64% of breast cancer patients undergo breast-conserving surgery, while 36% undergo mastectomy [3]. Often these procedures involve the prior placement of a guidewire to assist in tumour localisation during surgery. It is therefore important that breast surgeons can correctly localise and physically characterise breast cancer lesions on mammography in order to optimise treatment outcomes and recurrence rate.

Mammographic breast density (BD) refers to the proportion of fibro-glandular tissue to fatty tissue in the breast and is frequently associated with a radio-opaque appearance on mammograms [4]. The ACR Bi-RADS system divides this into 4 categories: A (<25% fibroglandular tissue or BD), B (25–50%), C (50–75%) and D (>75%). It has previously been reported that women with heterogeneously dense breasts and extremely dense breasts have higher breast cancer rates (1.48 to 1.62 per 1000 mammograms among women) compared to low women with low breast density (0.9 per 1000 examinations) [5]. Furthermore, women with more fatty and homogenous breast tissue have a higher detection sensitivity rate (80%) with mammography than women with heterogeneous breast mammographic appearance (67%) [6]. This is thought to be due to cancerous lesions being masked by breast tissue of higher density and heterogeneity and that tissue then also being superimposed [7, 8].

In Australia, the Breast Surgeons of Australia and New Zealand (BreastSurgANZ) provides first and second-year fellowship training for breast surgical trainees which includes a range of breast oncology, cancer surgery, breast reconstructive, melanoma and general surgical units [9]. This is offered in consultation and conjunction with the standard Royal Australasian College of Surgeons training. In terms of medical imaging training, the ultrasound workshop is the only radiological course in the compulsory training requirements for the Post Fellowship Training (PFT). A 2019 review of the value of the Graduate Certificate in Breast Surgery (GSBS) that is offered by the University of Sydney in collaboration with BreastSurgANZ did not include any commentary on the medical imaging curricula [10].

The Royal Australian and New Zealand College of Radiologists (RANZCR) provide a 5-year training programme and is structured in three major phases for radiology trainees. Radiology trainees must complete interpretation of at least 100 diagnostic mammograms and 500 screening mammogram cases during the three phases of their clinical radiology training. As such, there exists the opportunity to provide further radiological training to surgeons during PFT in order to improve their ability to correctly localise breast cancer lesions on mammography and use shared training and resources to improve interprofessional understanding of breast cancer appearances. Limited studies into the skills of general surgical trainees have shown that although they are often expected to use medical imaging to assist with surgical management, their interpretation skills are considerably below radiology trainees at a similar educational stage [11].

The first aim of this study is to investigate the performance of surgical trainees in breast cancer detection on digital mammograms. A secondary aim is to compare surgical trainees’ performance to that of radiology trainees in different levels of breast density, for the purpose of developing an understanding of the factors influencing performance between two groups of readers, and the suitability of shared cancer education resources. This comparison will quantify the potential benefits of integrating a mammography interpretation component into the surgical training programme. It will also establish a foundation for enhancing the expertise of breast surgeons, ultimately leading to improved outcomes in surgical procedures for breast cancer patients.

## Method

This was a retrospective observer performance study using digital mammogram reader data from BreastScreen Reader Assessment Strategy (BREAST) platform between 2020 and 2023 and received Human Research Ethics Committee (HREC) approval from the University of Sydney (2019/013). The two comparison groups that participated in this observational study were Australian breast surgical trainees and general radiology trainees, who each completed the same mammographic test set on the BREAST platform, which contained 20 mammogram cases consisting of 6 breast cancer cases. The test set consisted of 3 low BD cases (levels A and B) and 3 high BD (levels C and D) cancer cases plus 14 cancer-free cases (5 low BD and 9 high BD).

The cancer-free mammogram cases were confirmed by at least two senior radiologists with consensus reads in two rounds of normal screening, while the cancer cases were confirmed by breast tissue biopsy. Each case in the test set contained two mammographic views, a cranio-caudal (CC) and mediolateral oblique (MLO) for each side. The test set did not include mammogram cases with post-biopsy markers, surgical clips, or scars. The Cancer Institute New South Wales (CINSW) waived the need for obtaining informed consent from the patients whose anonymised mammograms were used in the test set. All reading examinations were performed after obtaining informed consent from participants.

Recruitment of surgical trainees was conducted by the unit coordinator of a Breast Surgery unit of study at the University of Sydney (as part of the Graduate Certificate of Breast Surgery (GCBS)), and test set readings of surgical trainees were conducted between 2020 and 2023. The data sample of radiology trainees was collected through the BREAST programme with data collection between 2022 and 2023. Recruitment of radiology trainees and surgical trainees was a voluntary process and yielded de-identified data. Radiology trainees include readers registered for reading the BREAST test set at their clinic or at the BREAST workshop which has similar reading environment.

The sample size included 18 surgical trainees (10 with <3 months experience and 8 with ≥3 months experience) and 32 radiology trainees (28 with <3 months experience and 4 with ≥3 months experience) who completed reading the same cases between 2022 and 2023. Each reader’s information was collected through an online demographic questionnaire embedded in the BREAST platform, including their current role, medical specialty (breast or other specialisation), time in current role (average 5 years for surgical trainees and average 3 years for radiology trainees), years reading mammograms (average 11 months for surgical trainees and average 2 months for radiology trainees), number of mammographic cases read per week, number of hours reading per week and completion of breast fellowship. All data were collected with de-identified ID numbers.

The participants were asked to read the mammograms in full resolution on diagnostic monitors and were required to localise all suspected breast lesions for each mammogram case and rate each lesion in accordance with the RANZCR Imaging Classification: 1—no significant abnormality (no marking on the mammogram), 2—benign, 3—indeterminate/equivocal, 4—suspicious, or 5—malignant. The participants’ responses were recorded by the online BREAST platform.

The performances of participants were calculated in term of case sensitivity, specificity, location sensitivity, receiver operating characteristics (ROC) area under curve (AUC) and jackknife alternative free response receiver operating characteristics (JAFROC). Case sensitivity is a true positive rate measuring the proportion of cancer cases that were correctly marked with a positive rating (3, 4, or 5). Specificity measures the free-cancer cases that were correctly identified cancer-free (rating 1 or 2). Location sensitivity refers to the proportion of each cancer lesion that was correctly localised and identified. It assesses the ability of readers to accurately localise each breast lesion. ROC AUC evaluates the readers ability to detect cancer cases and identify normal cases, while JAFROC considers the performances of the readers in cancer localization in association with normal reporting and their ratings [12]. The participants were given access to their scores and the answers of each case after completing the test set in the BREAST platform.

The performance metrics of participants were analysed, first through an overall comparison between the surgical trainees and radiology trainees on all cases. The performance of surgical trainees was then compared to the performance of radiology trainees on low-density mammograms and high-density mammograms separately. Following this, the performance of surgical trainees was compared to the performance of radiology trainees on for trainee groups with <3 months experience and ≥3 months experience, separately. Finally, the performances of surgical and radiology trainees were compared by simultaneously stratifying the breast density levels, trainee experience and mammogram density.

The groups were compared in terms of specificity, sensitivity, lesion sensitivity, ROC and JAFROC values, with lesion sensitivity determined by a localisation within the radius of the true cancer lesion which was recorded based on the radiology and pathological reports. All cancer cases had only one actionable cancer. XXX statistical tests were used to for the comparisons. All statistical analyses were conducted with SPSS, and *P* value < 0.05 was considered significant.

## Results

### Performances of Radiology Trainees and Surgical Trainees in All Cases

**Fig. 1:**
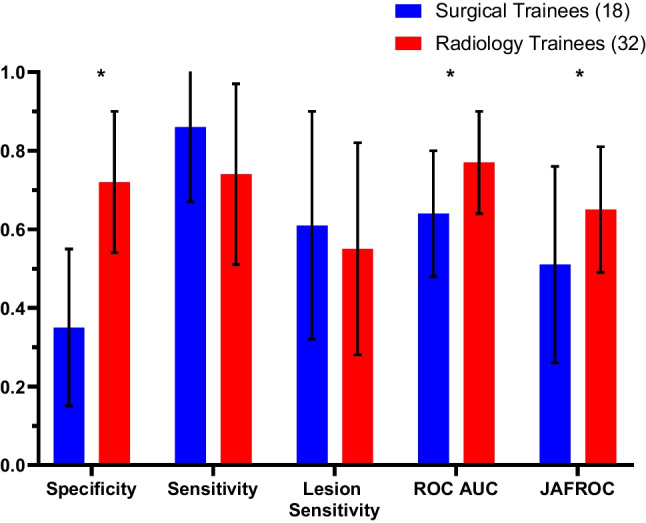
Performance of radiology trainees compared to surgical trainees in identifying cancer and normal cases. The groups were compared in terms of specificity, sensitivity, lesion sensitivity, ROC and JAFROC values (*P value < 0.05)

Overall, radiology trainees performed significantly better than surgical trainees in terms of specificity (0.72 vs. 0.35; *P* < 0.01), ROC AUC (0.77 vs. 0.64; *P* < 0.01) and JAFROC (0.65 vs. 0.51; *P* = 0.05). No significant differences were observed between the surgical and radiology trainees in sensitivity or lesion sensitivity (Fig. 1).

### Performances of Radiology Trainees and Surgical Trainees in Low Breast Density Mammograms

**Fig. 2:**
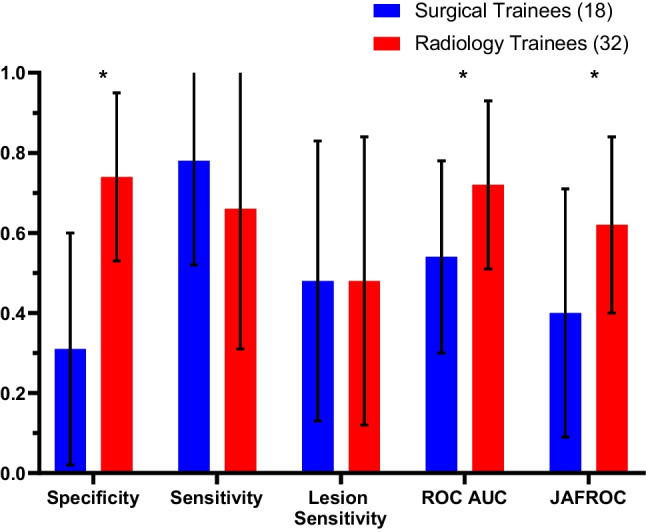
Performance of surgical trainees on low-density mammograms compared to radiology trainees on low-density mammograms in identifying cancer and normal cases. The groups were compared in terms of specificity, sensitivity, lesion sensitivity, ROC and JAFROC values (*P value < 0.05)

On low-density mammograms, it was found that the radiology trainees performed better than the surgical trainees in terms of specificity (0.74 vs. 0.31; *P* < 0.01), ROC AUC (0.72 vs. 0.54; *P* = 0.01) and JAFROC (0.62 vs. 0.40; *P* = 0.01), whereas no significant differences were observed in sensitivity or lesion sensitivity (Fig. 2).

### Performances of Radiology Trainees and Surgical Trainees in High Breast Density Mammograms

**Fig. 3:**
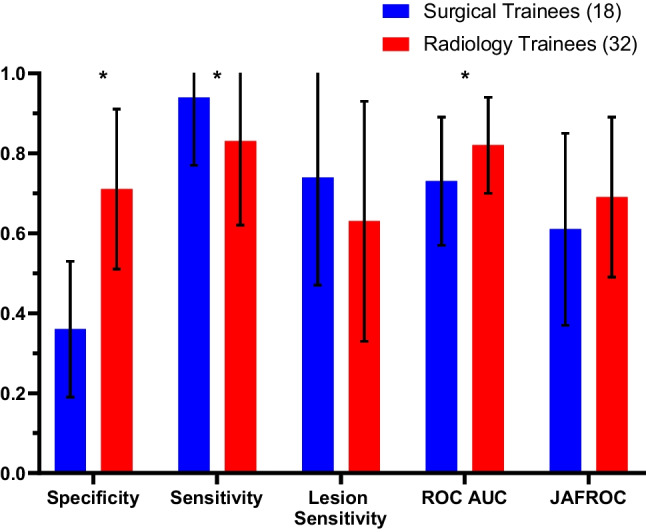
Performance of surgical trainees on high-density mammograms compared to radiology trainees on high-density mammograms in identifying cancer and normal cases. The groups were compared in terms of specificity, sensitivity, lesion sensitivity, ROC and JAFROC values (*P value < 0.05)

A similar pattern was found on mammograms with high breast density as the radiology trainees outperformed the surgical trainees in terms of specificity (0.71 vs. 0.36; *P* < 0.01) and ROC AUC (0.82 vs. 0.73; *P* = 0.05). However, the surgical trainees performed better than radiology trainees in terms of sensitivity (0.94 vs. 0.83; *P* = 0.03). No significant differences were observed in lesion sensitivity or JAFROC (Fig. 3).

### Performances of Surgical Trainees Compared with Radiology Trainees Who Had Less than 3 Months of Experience Reading Mammograms

**Fig. 4:**
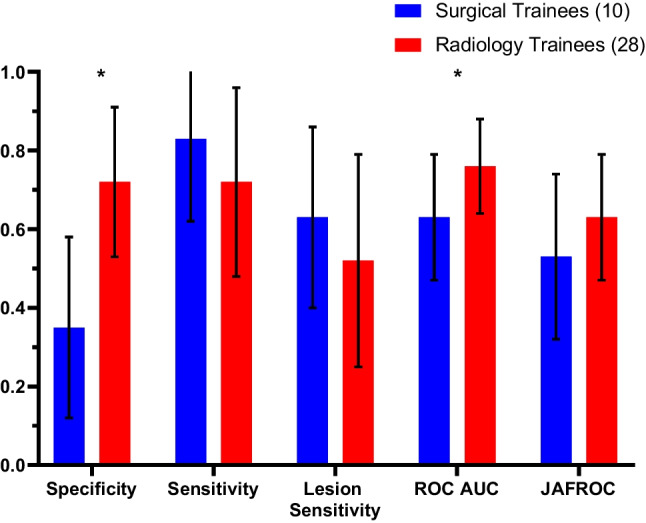
Performance of surgical trainees compared to radiology trainees in identifying cancer and normal cases on mammograms. Both surgical and radiology trainees had <3 months of experience in reading mammograms. The groups were compared in terms of specificity, sensitivity, lesion sensitivity, ROC and JAFROC values (*P value < 0.05)

For trainees with <3 months of experience reading mammograms (*N* = 28 radiology and *N* = 10 surgical), it was found that the radiology trainees performed better than the surgical trainees in terms of specificity (0.72 vs. 0.35; *P* < 0.01) and ROC AUC (0.76 vs. 0.63; *P* = 0.02), whereas no significant differences were observed in sensitivity, lesion sensitivity or JAFROC (Fig. 4). Furthermore, radiology trainees performed significantly better than the surgical trainees on low breast density cases in terms of specificity (0.74 vs. 0.36; *P* < 0.01) only, and on the high-density cases, radiology trainees performed significantly better than the surgical trainees in both specificity (0.71 vs. 0.34; *P* < 0.01) and ROC (0.82 vs. 0.71; *P* = 0.04).

### Performances of Surgical Trainees Compared with Radiology Trainees Who Had ≥3 Months of Experience Reading Mammograms

**Fig. 5:**
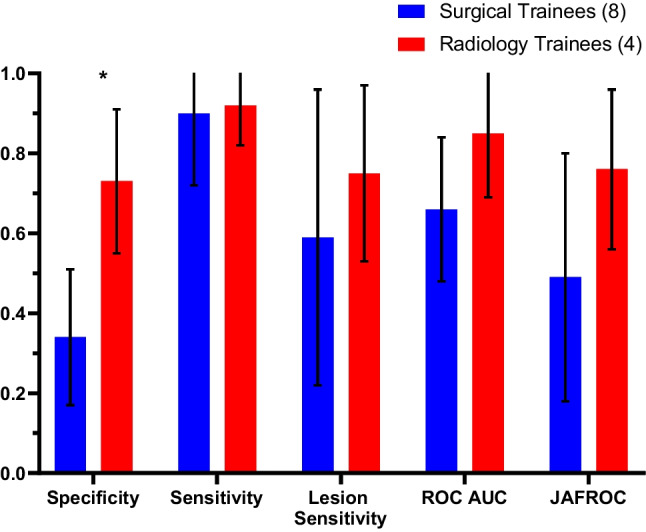
Performance of surgical trainees compared to radiology trainees in identifying cancer and normal cases on mammograms. Both surgical and radiology trainees had ≥3 months of experience in reading mammograms. The groups were compared in terms of specificity, sensitivity, lesion sensitivity, ROC and JAFROC values (*P value < 0.05)

The radiology trainees with ≥3 months of experience (*N* = 8 radiology and *N* = 4 surgical) performed significantly better than the surgical trainees in terms of specificity (0.73 vs. 0.34; *P* = 0.01), whereas no significant differences were observed in sensitivity, lesion sensitivity, ROC AUC or JAFROC (Fig. 5). In addition, the radiology trainees outperformed the surgical trainees on low-density cases, in terms of specificity (0.80 vs. 0.25; *P* < 0.01) as well as ROC (0.88 vs. 0.53; *P* = 0.05), while on the high-density cases, radiology trainees performed significantly better than the surgical trainees in specificity (0.69 vs. 0.39; *P* = 0.03) only (Table 1).

**Table 1:** Performance of surgical trainees compared to radiology trainees in identifying cancer and normal cases on mammograms. The groups were compared in terms of specificity, sensitivity, lesion sensitivity, ROC and JAFROC values. The results for these outcomes were pooled based on both mammogram density (low vs. high) and trainee experience (<3 months vs. ≥3 months)

		Specificity	Sensitivity	Lesion sensitivity	ROC	JAFROC
Surgical trainees, low density, <3 months exp.	*N*	10	10	10	10	10
	Mean	0.36	0.73	0.50	0.55	0.44
	SD	0.30	0.26	0.28	0.23	0.27
	Median	0.30	0.67	0.50	0.57	0.37
Radiology trainees, low density, <3 months exp.	*N*	28	28	28	28	28
	Mean	0.74	0.62	0.45	0.70	0.60
	SD	0.22	0.36	0.37	0.21	0.22
	Median	0.80	0.67	0.67	0.78	0.63
	*P* value	<0.01*	0.44	0.77	0.06	0.07
Surgical trainees, high density, <3 months exp.	*N*	10	10	10	10	10
	Mean	0.34	0.93	0.77	0.71	0.60
	SD	0.21	0.21	0.22	0.16	0.20
	Median	0.39	1.00	0.67	0.75	0.63
Radiology trainees, high density, <3 months exp.	*N*	28	28	28	28	28
	Mean	0.71	0.82	0.60	0.82	0.67
	SD	0.21	0.21	0.31	0.10	0.19
	Median	0.72	1.00	0.67	0.82	0.70
	*P* value	<0.01*	0.08	0.12	0.04*	0.29
Surgical trainees, low density, ≥3 months exp.	*N*	8	8	8	8	8
	Mean	0.25	0.83	0.46	0.53	0.35
	SD	0.30	0.25	0.43	0.27	0.37
	Median	0.20	1.00	0.50	0.43	0.25
Radiology trainees, low density, ≥3 months exp.	*N*	4	4	4	4	4
	Mean	0.80	0.92	0.67	0.88	0.74
	SD	0.16	0.17	0.27	0.13	0.19
	Median	0.80	1.00	0.67	0.88	0.70
	*P* value	0.02*	0.61	0.43	0.05*	0.09
Surgical trainees, high density, ≥3 months exp.	*N*	8	8	8	8	8
	Mean	0.39	0.96	0.71	0.77	0.62
	SD	0.13	0.12	0.33	0.16	0.29
	Median	0.39	1.00	0.83	0.80	0.67
Radiology trainees, high density, ≥3 months exp.	*N*	4	4	4	4	4
	Mean	0.69	0.92	0.83	0.84	0.80
	SD	0.19	0.17	0.19	0.20	0.22
	Median	0.72	1.00	0.83	0.90	0.81
	*P* value	0.03*	0.60	0.58	0.35	0.27

### Performances of Surgical Trainees and Radiology Trainees with Different Experience Reading Mammograms

For surgical trainees with <3 months of experience reading mammograms, their performance on high-density mammograms was significantly better than on low-density mammograms in terms of sensitivity (0.93 vs. 0.73; *P* = 0.03), lesion sensitivity (0.77 vs. 0.50; *P* = 0.01) and ROC (0.71 vs. 0.55; *P* = 0.55). For surgical trainees with ≥3 months of experience, their scores on high-density mammograms were significantly better than on low-density mammograms in terms of lesion sensitivity (0.71 vs. 0.46; *P* = 0.03), ROC (0.77 vs. 0.53; *P* = 0.04) and JAFROC (0.62 vs. 0.35; *P* = 0.03) (Table 1).

Furthermore, for radiology trainees with <3 months of experience reading mammograms, it was found that they performed significantly better on high-density mammograms than on low-density mammograms in terms of sensitivity (0.82 vs. 0.62; *P* = 0.01) and ROC (0.82 vs. 0.70; *P* = 0.01), whereas for the trainees with ≥3 months of experience, no significant differences were found in any of the outcomes (also see Table 1).

## Discussion

This study, for the first-time, explored proficiencies of both radiology and surgical trainees in interpreting screening mammograms. With the ever-increasing significance of early breast cancer detection, this research is poised to offer critical insights into the competencies of future medical specialists that diagnose and perform breast surgery. By focusing on radiology and surgical trainees, it not only addresses an important aspect of specialised training but also has the potential to enhance patient outcomes through simulated experiences using real clinical cases.

The higher specificity of the radiology trainees compared to the surgical trainees (Fig. 1) is likely due to the surgical trainees having less exposure to negative mammography cases and are therefore they may be less experienced in identifying normal cases and variants. This is supported by the comparable performances of the radiology and surgical trainees in sensitivity and lesion sensitivity where no significant difference was found, which suggests that the surgical trainees are better able to identify and mark positive breast cancer lesions, likely because the majority of the mammograms being viewed by surgeons are cases that have already been identified as positive. This reflects the importance of correct lesion identification and localisation in breast surgical procedures. The ROC and JAFROC metrics represent the reader capacity to distinguish between normal and cancer cases (ROC) or cancer lesion locations (JAFROC). The elevated ROC AUC and JAFROC scores achieved by radiology trainees imply their increased confidence in correctly identifying normal cases and greater precision in cancer localization compared to their counterparts. Similar results were observed between surgical and radiology trainees with <3 months of experience and ≥3 months of experience, with the metrics being slightly more similar in the ≥3 months of experience group (Figs. 4 and 5 and Table 1), suggesting that performance metrics become more equal given time and practise for both groups.

In comparing performance between trainees on high-density and low-density cases, it was somewhat surprising that both of the surgical and radiology trainees with <3 months of experience performed slightly better on the high-density mammogram cases in terms of sensitivity, lesion sensitivity and ROC. Such findings were unexpected, as high breast density mammograms are typically considered more challenging to interpret compared to their low-density counterparts as they frequently occlude cancerous lesions [13, 14]. This might suggest that novice trainees may adapt more quickly to the complexities posed by high-density mammograms, possibly due to their fresh perspective and adaptability in learning new skills. Further investigation into the underlying factors contributing to this phenomenon could yield valuable insights for training programmes and improve the overall proficiency of trainees in reading mammograms of varying densities.

The findings of this study shed light on a valuable perspective regarding the integration of medical imaging education, namely, mammography but also other key modalities too, into the surgical training programme. As surgical excision of breast cancers is common and a complex task, especially when some cancers like calcifications and architectural distortions are non-palpable, the medical imaging education would enhance surgical trainees’ ability to distinguish the visual characteristics of normal and cancerous tissue as seen on mammograms. This is a crucial skill as it contributes to informed decision-making before and during surgical procedures. By improving the proficiency in identifying normal and cancerous cases, surgical trainees would be better equipped to collaborate effectively with radiology trainees, radiologists and oncologists, ultimately leading to more comprehensive patient care. Multidisciplinary meetings (MDT) in the management of breast cancer have shown improved confidence in medical specialities for radiology participants and an international review of MDT report that 97% of participants believe improved clinical care for patients was achieved [15, 16].

This insight also emphasises the importance of tailoring training programmes to the specific needs of trainees and aligning the curriculum with the demands of future clinical practise. BREAST has been shown to improve clinical performance of radiologists, and a similar education platform can be tailored to surgeons to help assist with tumour margin detection, localisation of non-palpable lesions and correct identification of benign or normal variants with the breast that may also contain malignant features. Furthermore, immediate feedback mechanisms, such as those provided by BREAST, are very important as it has been shown to improve subsequent performance in reviewing medical images [17]. Eid et al. (2019) also call for greater radiology training into non-radiology curricula to improve the recognition of clinically significant pathologies where medical imaging is the prime diagnostic test [18].

## Conclusion

The ability to recognise breast cancer appearances on mammograms is a critical skill for both surgical and radiology trainees, with the medical images proving a guidance pre-surgery and is influential in decision-making about hook wire localisation and surgical entry point. Surgical trainee’s performance was equal to that of radiology trainees in lesion sensitivity and sensitivity, but significantly below in specificity. Online high-fidelity self-testing platforms that use real cases are the ideal cancer education environment to benchmark radiology interpretation skills, which has a direct relationship to patient care in terms of surgical planning. Breast surgical training in Australia, to this point in time, had not had a considerable amount of corresponding medical imaging training, and this study has identified a need to provide cancer education resources that challenge trainees in a range of patient presentations, including mammography cases with high breast density. The study has also highlighted the importance of simulated cancer detection platforms as a mechanism for increasing experience when case reading numbers are low.

## Data Availability

Data availability can be requested from the corresponding author.
